# Kinematic Factors Associated with Hitting Hurdles During the Initial Phase of a 110-m Hurdle Race

**DOI:** 10.2478/hukin-2022-0048

**Published:** 2022-09-08

**Authors:** Ryo Iwasaki, Hironari Shinkai, Hiroyuki Nunome, Nobuyuki Ito

**Affiliations:** 1 Faculty of Sports and Health Science, Fukuoka University, Fukuoka, Japan; 2 The United Graduate School of Education, Tokyo Gakugei University, Tokyo, Japan; 3 Faculty of Education, Tokyo Gakugei University, Tokyo, Japan; 4 Faculty of Education, Yokohama National University, Kanagawa, Japan

**Keywords:** motion analysis, athletics, kinematics

## Abstract

This study aimed to clarify the kinematic factors for the cause and effect of hitting hurdles during the initial phase of a 110-m hurdle run. Nine experienced male hurdlers participated in this study (body height: 1.74 ± 0.04 m, body mass: 67.4 ± 5.9 kg, age: 20.2 ± 1.4 years, personal best: 15.21 ± 0.47 s, seasonal best: 15.33 ± 0.55 s). Hurdlers undertook 12 trials of the initial phase of hurdling from the start to the second hurdle landing. Dual-sided sagittal plane motion was obtained from images from two high-speed cameras operating at 120 Hz. One ‘hit’ trial which had the largest horizontal displacement of markers fixed on the hurdle and one ‘non-hit’ trial which had the fastest time of hurdle clearance were extracted for each participant. Kinematic variables were compared between the two trials. Significantly lower height of the whole-body centre of mass at the take-off was found as a possible cause of hitting hurdles, caused by insufficient swing-up of the lead leg thigh. In contrast to conventional understanding, take-off velocity, take-off distance and the take-off angle were comparable between the ‘hit’ trial and ‘non-hit’ trial. Regarding the effect of hitting hurdles, it was observed that running velocity during hurdling was not substantially reduced. However, several characteristic movements were identified that might induce inefficient motion to re-accelerate running velocity during the following landing steps.

## Introduction

The 110-m hurdles is a unique athletic event that combines sprinting and hurdle clearance. Top-level 110-m hurdlers are required to have not only good sprint ability, but also to have efficient hurdle clearance technique (Bedini, 2012). During hurdle clearance, a hurdler must have an asymmetric leg movement: the lead leg first clears the hurdle with a fully extended posture and the trail leg subsequently follows to clear the hurdles with an abducted and bent knee posture. As hurdle clearance is an essential element to improve performance (Coh and Iskra, 2012), many researchers have conducted biomechanical studies focusing on clearance techniques (Coh and Iskra, 2012; McDonald, 2002; McDoland and Dapena, 1991; McLean, 1994). During the take-off phase, maintaining horizontal velocity as high as possible is important because it decreases owing to the necessary increase in the vertical component of velocity required for hurdle clearance regardless of the competitive level (McDonald, 2002). Moreover, decreases in running speed in the last half of the race were found to be due to insufficient hurdle clearance technique rather than fatigue (Salo and Scarborough, 2006).

It is logical that hitting hurdles may reduce the running speed of hurdlers. In the 110-m hurdles event, as the height of the hurdle (1.067 m) is the highest among the hurdle events, hitting hurdles occurs more frequently than in other hurdle events such as the women’s 100-m hurdles (0.84 m) and the men’s 400-m hurdles (0.914 m).

In general, when hitting hurdles there is a loss of balance and it then becomes difficult to prepare for optimal clearance of the subsequent hurdle (Salo and Scarborough, 2006). One typical case was reported in the International Association of Athletics Federations (IAAF) World Championships in Athletics in 2017 where a hurdler in the third place until the seventh hurdle increased touchdown time by hitting the eighth hurdle and then suddenly dropped to the fourth place (Pollitt et al., 2018). On the other hand, hurdlers sometimes win the race and beat personal records even if they hit several hurdles. Another typical case can be observed from the IAAF World Championships in Athletics in 2009; the winner of the race hit the first hurdle, but achieved a personal record (Graubner and Nixdorf, 2011). These conflicting observations suggest that it is still unclear whether hitting hurdles itself has a substantial impact on performance. Hitting hurdles is generally considered to be caused by the hurdler being too close to the hurdle at the take-off or having a low take-off angle and also a low take-off velocity. Iwasaki et al. (2020) reported brief findings regarding hitting hurdles. However, there are not enough data to support common coaching assumptions. Scientific research on the cause and effect of hitting hurdles would bring new insight to coaching. Thus, this study was designed to clarify the kinematic factors related to the cause of hitting hurdles and to examine the effect of hitting hurdles on running velocity during the initial phase of the 110-m hurdles race. With regard to these aims, we set two hypotheses: 1) conventional technique factors including a closer position of the hurdler to the hurdle at the takeoff, and a low take-off angle and take-off velocity would explain why hurdlers hit hurdles, and 2) hitting hurdles itself has little impact on running velocity.

## Methods

### Participants

Nine experienced male 110-m hurdlers including four decathletes participated in this study (body height: 1.74 ± 0.04 m, body mass: 67.4 ± 5.9 kg, age: 20.2 ± 1.4 years, personal best: 15.21 ± 0.47 s, seasonal best: 15.33 ± 0.55 s). They were members of the university’s track and field team who trained 5–6 days per week for > 5 years. Prior to participation, all athletes received an explanation of the aims of the study and the experimental protocol and provided informed consent. The study was conducted in compliance with the Declaration of Helsinki for Human Research and an Institutional Research Ethics Committee approved this study.

### Measurements

According to the official standard for the 110-m hurdles, the distance between the first and the second hurdle was set at 9.14 m. Participants were asked to perform a maximal effort hurdle run from the start until after the second hurdle clearance. All participants repeated this 12 times with sufficient rest between each trial so that one clear trial without hitting the first hurdle and one trial where the hurdler hit the first hurdle were obtained. Participants were not instructed regarding hitting hurdles. Therefore, all trials were random data. Twenty-four markers were attached to participants, including the head, upper sternum and right and left ears, shoulders, elbows, wrists, hands, lower-ribs, hips, knees, ankles, heels and toes. Two additional markers were attached to both edges of the first hurdle’s bar. Images of the sagittal plane motion of all trials were obtained at 120 Hz using two electrically synchronized high-speed cameras (HAS-L1, Ditect, Japan) placed on either side of the first hurdle.

### Procedures

All body and hurdle markers were digitized using motion analysis software (Frame-DIAS V, DKH, Japan). The coordinates of the markers obtained by two-dimensional Direct Linear Transformation (Walton, 1981) were smoothed using a 4th order Butterworth low pass digital filter with cut-off frequencies of 7.1 - 14.3 Hz. Cut-off frequencies were determined from residual analysis (Winter, 2009). A whole-body kinematic model consisting of 15 rigid segments (head, upper trunk, lower trunk, upper arms, lower arms, hands, thighs, shanks and feet) was used for analysis. The centre of mass (CoM) location was determined using the data of young living Japanese athletes by Ae et al. (1992) based on the procedure first described by Jensen (1978).

We analyzed the first hurdle clearing motion from the beginning of the take-off step before the hurdle to the instant of landing of the step after the hurdle (Figure 2). Of all 12 recorded trials, one non-hit trial (NHT) which had the fastest elapsed time of hurdle clearance for each participant and one hit trial (HT) which had the largest horizontal displacement of the hurdle markers were analyzed.

**Figure 1:**
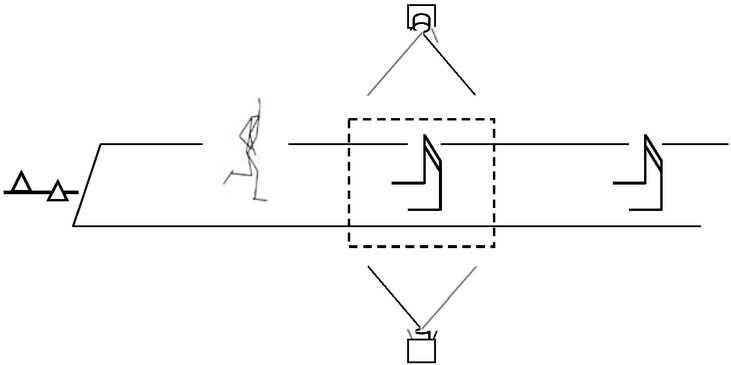
Experimental setup The area denoted by the dotted line corresponds to the approximate analysis phase

**Figure 2:**
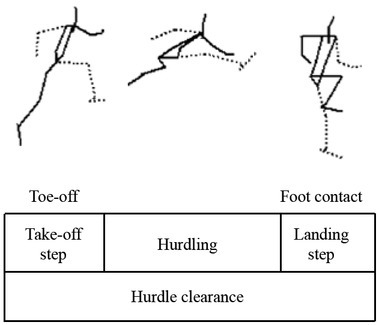
Definition of the hurdle clearance phase

**Figure 3:**
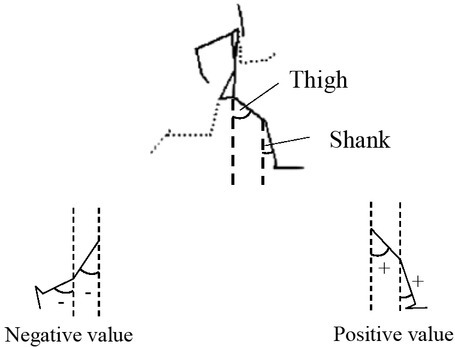
Definition of lower limb movements

### Hurdling variables

Kinematic variables regarding hurdle clearance were calculated as follows: 1) hurdling horizontal distances; a) take-off distance which was defined as the horizontal distance from the toe of the trail leg to the hurdle at the take-off, and b) landing distance which was defined as the horizontal distance from the hurdle to the toe of the lead leg at landing, 2) hurdle clearance time; time spent on hurdle clearance (from the instant of toe-off of the trail leg to the instant of foot contact of the lead leg), 3) take-off angle; the average angle between the horizontal and the line connecting the whole-body CoM from the instant of trail leg foot release to 10 frames later, 4) takeoff horizontal and vertical velocities; the horizontal and vertical velocities of the whole-body CoM at the instant of the trail leg toe-off, 5) landing horizontal and vertical velocities; the horizontal and vertical velocity of the CoM at the instant of the lead leg foot contact, 6) amount of velocity reduction; the difference between the landing horizontal velocity and the take-off horizontal velocity, 7) CoM height; the height of the whole-body CoM at the instant of the trail leg toe-off, and 8) contact time of the breaking phase at landing; the time when the distance from the toe of the lead leg to the CoM was negative at the landing step.

### Lower limb movements

Lower limb movements through the analyzed portion were calculated as angles and angular velocities. In accordance with a previous study (Shibayama et al., 2011), two angles were defined as segmental angles between the vertical and the thigh segment (thigh angle) and the shank segment (shank angle) (Figure 3). The angles at the instant of the trail leg toe-off and the lead leg foot contact, and maximum and minimum angular velocities during the supporting phase of the take-off step and the landing step, were calculated.

### Statistical analysis

All variables are presented as mean ± standard deviation (SD). Data normality was analyzed using the Shapiro–Wilk test. To assess the differences, Student’s paired *t*-tests for parametric data (Shapiro–Wilk test, *p* > 0.05) were used. To evaluate differences in landing horizontal velocity, the lead leg shank angle at the landing step, the minimum angular velocity of the thigh of the trail leg in the take-off step and contact time of the breaking phase at landing, Wilcoxon signed–rank tests were used because the data normality was not confirmed (Shapiro–Wilk test, *p* < 0.05). To describe the typical error, the coefficient of variation (CV) was calculated to determine the absolute variability between the intra-conditions. The level of significance was set at *α* < 0.05. To control the family-wise error rate, the alpha level of each *t*-test was adjusted with the Holm’s method. Cohen’s *d* was used to describe the effect size (Cohen, 1992). Values of < 0.2, 0.2-0.5, and > 0.5 were considered small, medium, and large, respectively. All statistical analyses were performed using EZR (version 1.37; Saitama Medical Center, Jichi Medical University; Kanda, 2013), which is a graphical interface for R (The R Foundation for Statistical Computing, Vienna, Austria) designed to add statistical functions frequently used in biostatistics.

## Results

### Hurdling variables

Table 1 shows the hurdling variables. CoM height at the instant of the trail leg toe-off was significantly lower in HT than in NHT (*p* = 0.009, *d* = 0.56, CV: NHT = 2.7%, HT = 2.4%). No marked differences were observed in the other hurdling variables between NHT and HT.

**Table 1:** Differences in hurdling variables

	NHT	HT
Hurdling horizontal distance (m)	3.91 ± 0.17	3.89 ± 0.11
Take-off horizontal distance (m)	1.91 ± 0.19	1.93 ± 0.20
Landing horizontal distance (m)	2.00 ± 0.14	1.96 ± 0.18
Hurdle clearance time (s)	0.46 ± 0.04	0.45 ± 0.03
Take-off angle (deg)	15.5 ± 1.6	15.2 ± 1.9
Take-off horizontal velocity (m/s)	7.16 ± 0.28	7.24 ± 0.28
Take-off vertical velocity (m/s)	2.34 ± 0.20	2.32 ± 0.19
Landing horizontal velocity (m/s)	6.92 ± 0.31	6.94 ± 0.19
Landing vertical velocity (m/s)	-2.21 ± 0.26	-2.09 ± 0.19
Amount of velocity reduction (m/s)	0.24 ± 0.13	0.30 ± 0.23
CoM height (m)	^*^1.15 ± 0.03	^*^1.13 ± 0.03
Contact time of the breaking phase at landing (s)	0.02 ± 0.01	0.03 ± 0.01

Values are expressed as the mean ± standard deviation. ^*^ Significant difference between NHT and HT (p < 0.05).

### Lower limb movements

Table 2 shows the angles at the instant of the trail leg toe-off and the lead leg foot contact. The lead leg thigh angle was significantly larger in NHT than in HT at the instant of the trail leg toe-off (*p* = 0.017, *d* = 0.70, CV: NHT = 5.9%, HT = 4.8%). The trail leg shank angle was significantly smaller in HT than in NHT at the instant of the lead leg foot contact; however, there was great variation in the intra-condition analysis (*p* = 0.011, *d* = 1.46, CV: NHT = 58.7%, HT = 35.9%). No marked differences were observed in the other angles between NHT and HT.

**Table 2:** Differences in lower limb angles

		Take-off step		Landing step	
		
		Foot release		Foot contact	
		NHT	HT	NHT	HT
Thigh	Lead (deg) leg	90.6 ± 5.4	^*^87.2 ± 4.2	16.7 ± 3.1	17.7 ± 3.6
	Trail (deg) leg	-13.9 ± 5.9	-14.6 ± 5.7	102.2 ± 5.7	91.6 ± 7.4
Shank	Lead (deg) leg	7.0 ± 11.8	-1.0 ± 7.6	2.9 ± 1.5	4.4 ± 4.7
	Trail (deg) leg	-32.0 ± 3.3	-32.4 ± 3.9	^*^-22.3 ± 13.1	^*^-43.2 ± 15.5

Values are expressed as the mean ± standard deviation. ^*^ Significant difference between NHT and HT (p < 0.05).

Table 3 shows the angular velocities of the lead leg and the trail leg in the take-off step and the landing step. No marked differences were observed in the thigh angular velocities of the lead leg and the trail leg between NHT and HT. The maximum angular velocity of the shank of the lead leg in the landing step was significantly smaller in HT than in NHT, however, there was great variation in the intra-condition analysis (*p* = 0.019, *d* = 0.75, CV: NHT = 27.1%, HT = 40.5%).

**Table 3:** Differences in lower limb angular velocities

		Take-off step				Landing step			
		
		Maximum		Minimum		Maximum		Minimum	
		NHT	HT	NHT	HT	NHT	HT	NHT	HT
Thigh	Lead leg	992.1	996.7	291.2	324.3	88.9	57.3	-524.1	-547.5
	(deg/s)	± 65.4	± 66.6	± 98.8	±82.9	± 113.7	± 113.3	± 46.6	± 40.7
	Trail leg	-59.4	-80.9	-613.2	-634.9	-34.3	14.1	-596.5	-510.2
	(deg/s)	± 50.1	± 45.7	± 50.5	± 50.6	± 69.2	± 78.1	± 77.7	± 89.5
Shank	Lead leg	1284.7	1306.0	-498.7	-550.3	^*^-297.2	^*^-232.0	-727.8	-777.9
	(deg/s)	± 71.6	± 77.7	± 104.2	± 151.2	± 80.5	± 94.0	± 69.3	± 90.7
	Trail leg	-58.9	-26.1	-649.3	-675.5	461.0	594.0	-185.6	18.6
	(deg/s)	± 47.0	± 66.6	± 67.4	± 46.9	± 80.1	± 115.4	± 216.9	± 163.0

Values are expressed as the mean ± standard deviation. ^*^ Significant difference between NHT and HT (p < 0.05).

## Discussion

The aim of this study was to clarify the kinematic factors related to the cause of hitting hurdles and to examine the effect of hitting hurdles on running velocity during the initial phase of the 110-m hurdles. To the best of our knowledge, this is the first study which has explored quantitative factors that likely cause the hitting of hurdles and has described their effect on running velocity. The main findings were: (1) the main cause of hitting hurdles was insufficient height of the CoM at the instant of the trail leg toe-off, and (2) running velocity during hurdling was not substantially reduced by hitting hurdles. The first finding did not support our initial hypothesis regarding the cause of hitting hurdles. Conventional kinematic factors, a closer position to the hurdle at the take-off and a low take-off angle and take-off velocity of hurdlers did not explain why hurdlers hit hurdles. On the other hand, the second finding, that hitting hurdles itself had little impact on running velocity, supported our initial hypothesis regarding the effect of hitting hurdles.

Of the many hurdling variables investigated, only the CoM height at the instant of the take-off was different between the non-hit trial (NHT) and hit trial (HT) conditions. In contrast to the conventional understanding, hurdling variables such as take-off velocity, take-off distance and a take-off angle were comparable between the two conditions. These findings are supported by a previous study (Iwasaki et al., 2020). Theoretically, it is necessary for hurdlers to achieve an optimal CoM height during the takeoff step in order to clear the hurdles. As hurdlers attempt to minimize their vertical fluctuation during hurdle clearance, maintaining a high position of the CoM during the take-off step has been considered an important element (Coh, 2003). Therefore, it has been assumed that hurdlers hit hurdles due to their lower CoM height at the instant of the take-off. Several quantitative analyses have already stressed that CoM height at the take-off step is one of the most important factors for efficient execution of hurdle clearance (Amara et al., 2019; Coh and Iskra, 2012). In the present study, we succeeded in confirming the importance of CoM height to achieve clear hurdle clearance from a biomechanical perspective.

A fundamental hurdling technique is to swing the lead leg forward and upwards to increase CoM height and to clear the hurdle (McDonald and Dapena, 1991). Among the lower limb movements during the take-off step, the lead leg thigh angle at the instant of the trail leg toe-off was found to be significantly smaller in HT compared to NHT. It can be assumed that the smaller initial lead leg thigh angle would disturb the forward swing of the lead leg due to its greatest mass proportion off all lower limb segments. It was suggested that the insufficient upwards swing of the lead leg thigh may account for the lower CoM height in HT at the instant of the take-off.

Prior to this study, it was unknown whether hurdle hitting itself would substantially reduce running velocity during hurdle clearance. Iwasaki et al. (2020) reported that CoM horizontal velocity at the landing was not significantly different between the hit trial and the non-hit trial. Pollitt et al. (2018) suggested that hitting hurdles might not affect the resultant performance in a 110-m hurdles race. The findings of the present study support this previous finding; however, there is a possibility that hitting hurdles might induce inefficient motion for acceleration during the following landing step. During the landing step, the maximum angular velocity of the lead leg shank in HT was found to be significantly lower than that of NHT. As faster hurdlers tend to have a larger negative angular velocity of the lead leg shank through the landing step (Shibayama et al., 2011), it can be interpreted that a larger negative angular velocity of the lead leg shank would contribute towards a faster running velocity. Thus, it can be assumed that hitting the hurdle leads to a forced change in landing leg motion during the landing step and this change may disturb hurdlers’ ability to re-accelerate their running velocity. Landing technique has been recognized as one of the most important elements for sprint hurdling (Coh and Iskra, 2004; McLean, 1994). Even if hitting hurdles does not substantially reduce running velocity as it was found in this study, performance of subsequent running may be negatively affected if landing technique becomes inefficient for re-acceleration of the body. Therefore, it can be speculated that hitting hurdles has a negative impact on the following re-acceleration phase. However, the CV in the maximum angular velocity of the lead leg shank was high, thus the repeatability should be verified. Moreover, as the motions throughout the landing step were beyond the focus of the present study, further investigation of this phase of hurdling appears warranted.

Regarding the trail leg movements, the angle of the trail leg shank at the instant of the lead leg foot contact was significantly smaller in HT than in NHT. During the landing step, the trail leg movement is one of the most important features of technique (Coh, 2003). Salo and Scarborough (2006) argued that hitting hurdles put an athlete off balance. Thus, it can be speculated that hurdlers were unable to pull the trail leg forward sufficiently and/or found it difficult to maintain balance during the landing step after hitting hurdles. Therefore, it was suggested that hitting hurdles might disturb the trail leg movement. However, as the trail leg executes a complicated three-dimensional movement, it was not possible to describe it with high accuracy and repeatability in the present study and again this issue warrants future attention.

In the current study, the part of the body that actually hit the hurdle was not documented. The effects of hitting hurdles could be influenced by hitting with the lead leg versus the trail leg (Iwasaki et al., 2020) and this is a limitation of the current investigation. Another limitation of this study is the fact that the sample size was not sufficient to obtain an optimal statistical power. Future studies should verify the accuracy and repeatability of kinematic variables associated with hitting hurdles. Furthermore, we only focused on the first hurdle. Further investigation of hitting hurdles including other hurdles appears warranted.

In summary, we found that the main cause of hitting hurdles was a significantly lower height of the whole-body CoM at the take-off due to an insufficient upwards swing of the lead leg thigh. This finding was in contrast to the conventional understanding regarding the cause of hitting hurdles such as the take-off distance, the take-off angle and take-off velocity of the hurdler. As for the effect of hitting hurdles, we found that hitting hurdles did not substantially reduce running velocity during hurdling. However, several characteristic movements that might induce inefficient motion for re-accelerating running velocity during the following landing steps were observed. It was suggested that although hitting hurdles itself had little direct impact on running velocity, it may perhaps lead to inefficient, imbalanced running motions during the following hurdle interval.
